# Supplementary material for: The interactome of intact mitochondria by cross-linking mass spectrometry provides evidence for coexisting respiratory supercomplexes
Source: Mol Cell Proteomics. 2017 Dec 8;17(2):216–32. doi: 10.1074/mcp.RA117.000470 (PMC5795388; doi:10.1074/mcp.RA117.000470)
Supplement: Supplemental Data [file supp_RA117.000470_133922_0_supp_23978_fzffwf.zip › spectra_annotation/mito_spectra_annotation/359-1-23-1-4-1.pdf]

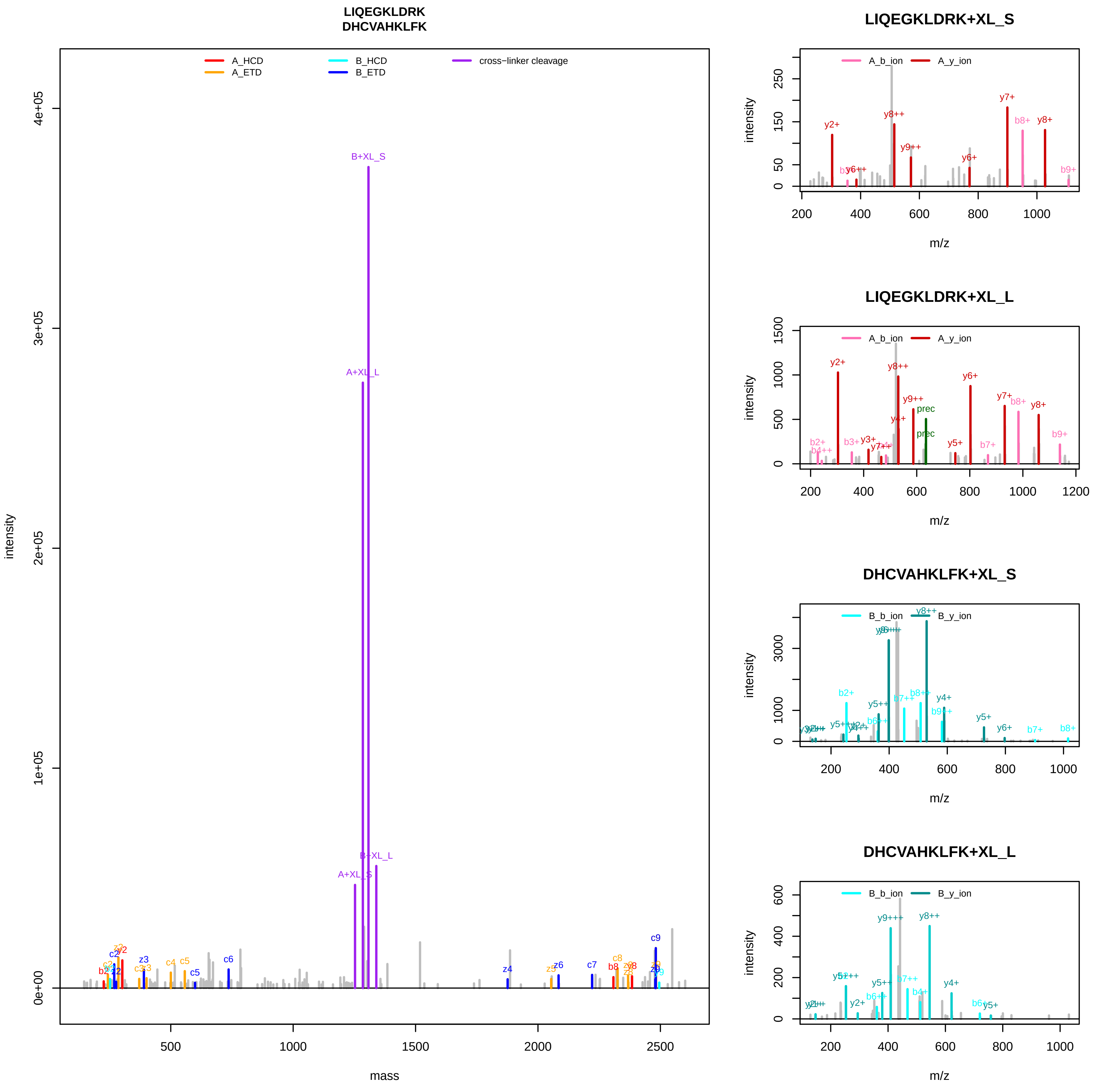
LIQEGKLDRK
DHCVAHKLFK
A_HCD
A_ETD
B_HCD
B_ETD
cross−linker cleavage
B+XL_S
A+XL_L
B+XL_L
A+XL_S
intensity
mass
4e+05
3e+05
2e+05
1e+05
0e+00
500
1000
1500
2000
2500
c2
c3
c5
c6
c7
c8
c9
z3
z4
z5
z6
b8
y8
LIQEGKLDRK+XL_S
A_b_ion
A_y_ion
y2+
y8++
y9++
y6+
y7+
b8+
y8+
b9+
m/z
LIQEGKLDRK+XL_L
y2+
y8++
y6+
y9++
prec
y7+
b8+
y8+
b9+
y5+
b7+
DHCVAHKLFK+XL_S
B_b_ion
B_y_ion
y8++
b2+
y4+
y5+
y6+
b7+
b8+
DHCVAHKLFK+XL_L
y9+++
y8++
y4+
y2+
y5+
